# Risk of early progression according to circulating *ESR1* mutation, CA-15.3 and cfDNA increases under first-line anti-aromatase treatment in metastatic breast cancer

**DOI:** 10.1186/s13058-020-01290-x

**Published:** 2020-05-28

**Authors:** Florian Clatot, Anne Perdrix, Ludivine Beaussire, Justine Lequesne, Christelle Lévy, George Emile, Michael Bubenheim, Sigrid Lacaille, Céline Calbrix, Laetitia Augusto, Cécile Guillemet, Cristina Alexandru, Maxime Fontanilles, David Sefrioui, Lucie Burel, Sabine Guénot, Doriane Richard, Nasrin Sarafan-Vasseur, Frédéric Di Fiore

**Affiliations:** 1 Department of Medical Oncology, Centre Henri Becquerel, Rouen, France; 2 Normandie Univ, UNIROUEN, Inserm U1245, IRON group, Rouen University Hospital, Normandy Centre for Genomic and Personalized Medicine, Rouen, France; 3 Department of Biopathology, Centre Henri Becquerel, Rouen, France; 4 Clinical Research Unit, Centre Henri Becquerel, Rouen, France; 5 Institut Normand du Sein, Centre François Baclesse, Caen, France; 6 Department of Clinical Research and Innovation, Rouen University Hospital, Rouen, France

**Keywords:** ESR1 mutation, Breast cancer, Circulating DNA, CA-15.3, Cell-free DNA, Aromatase inhibitor

## Abstract

**Background:**

Endocrine therapy is recommended as a first-line treatment for hormone receptor-positive metastatic breast cancer (HR+MBC) patients. No biomarker has been validated to predict tumor progression in that setting. We aimed to prospectively compare the risk of early progression according to circulating *ESR1* mutations, CA-15.3, and circulating cell-free DNA in MBC patients treated with a first-line aromatase inhibitor (AI).

**Methods:**

Patients with MBC treated with a first-line AI were prospectively included. Circulating biomarker assessment was performed every 3 months. The primary objective was to determine the risk of progression or death at the next follow-up visit (after 3 months) in case of circulating *ESR1* mutation detection among patients treated with a first-line AI for HR+MBC.

**Results:**

Overall, 103 patients were included, and 70 (68%) had progressive disease (PD). Circulating *ESR1* mutations were detected in 22/70 patients with PD and in 0/33 patients without progression (*p* < 0.001). Among the *ESR1*-mutated patients, 18/22 had a detectable mutation prior to progression, with a median delay of 110 days from first detection to PD. The detection of circulating *ESR1* mutations was associated with a 4.9-fold (95% CI 3.0–8.0) increase in the risk of PD at 3 months. Using a threshold value of 25% or 100%, a CA-15.3 increase was also correlated with progression (*p* < 0.001 and *p* = 0.003, respectively). In contrast to *ESR1*, the CA-15.3 increase occurred concomitantly with PD in most cases, in 27/47 (57%) with a 25% threshold and in 21/25 (84%) with a 100% threshold. Using a threshold value of either 25% or 100%, cfDNA increase was not correlated with progression.

**Conclusion:**

The emergence of circulating *ESR1* mutations is associated with a 4.9-fold increase in the risk of early PD during AI treatment in HR+MBC. Our results also highlighted that tracking circulating *ESR1* mutations is more relevant than tracking CA-15.3 or cfDNA increase to predict progression in this setting.

**Trial registration:**

ClinicalTrials*.*gov, NCT02473120. Registered 16 June 2015—retrospectively registered after one inclusion (first inclusion 1 June 2015)

## Introduction

Tumor monitoring under treatment is currently based on clinical evaluation and imaging. In this context, the identification of early markers correlated with response to treatment is warranted to make real-time therapeutic adaptations. In metastatic breast cancer (MBC), an abnormal level or an elevation of CA-15.3 contributes to identifying tumor progression in conjunction with imaging, history of disease, and clinical course [1]. Until now, evidence has been too low to recommend the use of CA-15.3 instead of conventional follow-up.

In this context, liquid biopsy offers new perspectives for the real-time monitoring of tumor response under treatment. It has been shown that circulating tumor cells (CTCs) can be isolated in plasma, with a high concentration of CTCs correlated with poor prognosis [2]. Moreover, cell-free DNA (cfDNA) or circulating tumor DNA (ctDNA) has also been intensively evaluated in that setting. cfDNA is related to cell turnover, combining both normal DNA and tumoral DNA. Among cancer patients, the cfDNA level is correlated with disease stage [3] and is easily identifiable and quantifiable within all patients. Few data are available regarding the prognostic value of cfDNA in MBC. In a prospective study on 268 patients treated for MBC with first-line chemotherapy, cfDNA concentration was reported as an independent prognostic factor for both overall survival (OS) and progression-free survival (PFS) [4]. On the other hand, ctDNA detection, which is based on the determination of a circulating molecular alteration specific to tumoral DNA, has also shown promising results for disease monitoring.

To date, the first-line treatment of patients with hormonal receptor-positive MBC (HR+MBC) is based on aromatase inhibitors (AIs) combined with cdk4/6 inhibitors [5]. There are multiple mechanisms leading to resistance to endocrine therapy. Among them, mutations of the estrogen receptor gene (*ESR1*) have been associated with acquired resistance to AIs, with or without combination with cdk4/6 inhibitors [6, 7]. The detection of these mutations at progression with AIs is observed in 30–50% of cases and is associated with a poor outcome [6, 8]. Interestingly, these mutations can reliably be detected in blood, either by digital droplet PCR (ddPCR) [9–12] or by next-generation sequencing [12, 13]. Furthermore, the detection of circulating *ESR1* mutations several months before clinical progression has been observed in most patients [8, 14]. These criteria make *ESR1* mutations a potential biomarker for biological follow-up and therapeutic adaptation during AI treatment in advanced breast cancer.

To our knowledge, only one prospective study compared serial circulating biomarkers namely CTCs, ctDNA, and CA-15.3 in MBC [15]. Among 52 patients, 30 (58%) were finally analyzed for ctDNA mainly based on *PIK3CA* and *TP53* mutations. In these patients, ctDNA detection was found in at least one of the samples in 29 of the 30 patients (97%), and CA-15.3 was elevated in at least one of the samples in 21 of the 27 patients (78%). ctDNA had a better sensitivity and stronger association with tumor burden than CA-15.3 and CTCs. Moreover, while the emergence of circulating *ESR1* mutations has been identified as a predictive marker of AI resistance in HR+MBC, data comparing the effectiveness of ctDNA versus CA-15.3 or cfDNA in that setting are lacking. In this context, we aimed to assess CA-15.3, cfDNA, and circulating *ESR1* mutations to determine early progression in a prospective cohort of HR+MBC patients treated with a first-line AI.

## Patients and methods

### Study design

This study is based on an observational prospective cohort including HR+MBC patients treated with a first-line AI for MBC from June 2015. Due to the evolution of the knowledge regarding circulating *ESR1* mutations in 2016, an amendment regarding the objectives was accepted by regulatory agencies in January 2017, before the end of the inclusions and before any analysis. The inclusion criteria were as follows: women ≥ 18 years with MBC or non-operable locally advanced BC and treatment with AI initiated at inclusion or at least 6 months before with a documented non-progressive disease. Previous treatment for early BC with chemotherapy/tamoxifen/fulvestrant or AI was allowed with a time frame of 2 years between the last treatment and metastatic evolution. The exclusion criteria were participation in another clinical trial and hormone receptor-negative BC. The study was performed in the Henri Becquerel Cancer Centre, Rouen, France, and in the François Baclesse Cancer Centre, Caen, France. All patients provided informed consent, and the study was approved by an independent ethics committee. This prospective cohort was registered at www.clinicaltrials.gov (NCT02473120).

All included patients were followed up every 3 months with clinical examination and CT scan. Blood samples for circulating marker analysis were collected every 3 months using 2 tubes of 5 mL for ctDNA and cfDNA and using one tube of 5 mL for CA-15.3. A progressive disease (PD) was determined using the radiological evaluation by RECIST 1.1 [16] and the physician clinical evaluation. Each PD was confirmed by the Metastatic Breast Board of each center. Overall survival (OS) and progression-free survival (PFS) were defined as the time from AI initiation to death and the time from AI initiation to progression or death, respectively, and were censored at the last follow-up. Of note, OS and PFS at progression on AI were defined as the time from PD on AI to death and the time from PD on AI to progression or death, respectively. All patients with HER2-positive tumors were treated with anti-HER2 therapy.

### Plasma DNA extraction

Blood samples were collected in EDTA tubes and processed within 3 h after collection. First, the tubes were centrifuged at 1000*g* for 10 min at 4 °C. Then, plasma was transferred to micro-tubes and centrifuged at 16000*g* for 10 min at 4 °C. The plasma was then transferred to cryovials and stored at − 20 °C for 24 h, then at − 80 °C until analysis. DNA was extracted from 2 to 3 mL of plasma using the QIAamp®Circulating Nucleic Acid Kit (Qiagen, Hilden, Germany). Double-stranded DNA quantification was performed by a fluorometric method using the Quant-iT PicoGreen dsDNA Assay Kit (Invitrogen, Carlsbad, CA, USA) and a Twinkle LB970 microplate fluorometer (Berthold, BadWildbad, Germany).

### Circulating analyses

The CA-15.3 assay was performed by the BRAHMS Kryptor Plus compact controller using TRACE (Time-Resolved Amplified Cryptate Emission) technology. CA-15.3 was considered elevated when it was above the normal upper limit (30 U/mL). We considered a CA-15.3 increase as the first occurrence of a CA-15.3 increase of either 25% or 100%, between the CA-15.3 nadir and every 3 months of follow-up. Similar variation rates (25 and 100%) were also used for cfDNA analyses.

A droplet-based dPCR (ddPCR) platform (Qx200 ddPCR System, Bio-Rad Laboratories, Hercules, CA, USA) was used for the detection of mutant circulating DNA in plasma samples. The positive samples were discriminated from the negative samples by using two multiplex *ESR1* assays (dHsaMDXE91450042 *ESR1* Multiplex 1 targeting E380Q, D538G, Y537C, and L536R mutations and dHsaMDXE65719815 *ESR1* Multiplex 2 targeting S463P, Y537S, and Y537N mutations) (Bio-Rad Laboratories). The ddPCR reactions were performed in triplicate with 8 μL or 40 ng (depending on the amount) of cfDNA. A result was considered positive when 2 out of the 3 wells were positive. In the case of positive multiplex results, a simplex analysis for confirmation and identification of mutations was performed. Four nanograms of cfDNA was pre-amplified (9 cycles) using 12.5 μL of Q5 Hot Start High Fidelity Master Mix (New England Biolabs, Ipswich, MA, USA) and 0.7 μL of 20X each mutation’s assays (Thermo Fisher Scientific, Carlsbad, CA, USA) as previously described [8]. The following thermocycling PCR steps were used: 98 °C, 3 min; 9 cycles: 98 °C, 10 s; 60 °C, 3 min; 72 °C, 30 s and 72 °C, 2 min. ddPCR analyses were performed following the manufacturer’s recommendations using 2 μL of pre-amplified cfDNA. The total copy number for each sample was systematically between 200 and 2000 copies/μL per reaction. Negative control wells with no DNA were included in every run.

Background noise is the minimum concentration of the mutant allele that can be differentiated from a negative control. To assess the background noise of our method, the allele burden was measured in 11 cfDNA (for multiplex analysis) and 11 pre-amplified cfDNA extracted from healthy control EDTA plasma samples collected under the same conditions as the patient samples. In this study regarding the theoretical sample’s copy number and the background noise of each assay, a positive threshold of 0.1% was used.

All data were analyzed using QuantaSoft software (Bio-Rad) and were manually reviewed to provide a precise interpretation of the data points. The variant allele fraction (VAF) was defined as the proportion of mutant DNA copies relative to the sum of mutant and wild-type DNA copies obtained by ddPCR. Samples were considered mutated if at least two independent ddPCR analyses found a VAF above the mutation threshold. ddPCR analyses were all performed blindly from clinical data.

### Statistical analysis

The primary objective was to determine the risk of progression or death at the next follow-up visit (after 3 months) according to circulating *ESR1* mutation detection. The secondary objectives were to evaluate the risk of PD according to cfDNA and CA-15.3 increases and to determine the correlation between these biomarkers with progression or death. A predictive logistic regression model was thus performed, by using circulating *ESR1* mutation status assessed every 3 months during follow-up. Based on our previous data, we hypothesized that 30% of the patients would have an *ESR1* mutation detectable at progression. The observation of 56 progression events was required to detect at least a 2.4 risk ratio (RR) of progression or death until the next follow-up visit in the case of circulating *ESR1* mutation detection, assuming an alpha risk of 5% and 80% power. Comparisons between groups were made using the chi-squared test for categorical variables and the Wilcoxon-Mann-Whitney test for quantitative variables. Survival curves were estimated by the Kaplan-Meier method and compared using the log-rank test. We also reported the *p* value associated with the Cox model by considering CA-15.3 and cfDNA as continuous variables and assessing a possible monotone association between CA-15.3 (or cfDNA) and survival, without fixing a threshold. All statistical analyses were performed using R software version 3.0.1.

## Results

### Patient characteristics, clinical follow-up, and outcome

A total of 104 HR+MBC patients were prospectively included between June 2015 and August 2017. One patient was not considered for the analysis due to the lack of available plasma. The baseline characteristics of the 103 remaining patients are summarized in Table 1. Of note, 28 (27.2%) were already being treated with an AI at the time of inclusion. All patients had distant metastases except one who had tumor relapse with a deep invasion of the axillary fossa and permeation nodes. The median follow-up from AI initiation was 25 months (range 3–92). During that time, 70 patients (68%) experienced progression of the disease (PD), and 20 patients (19%) died. The median PFS and OS were 20.6 months and not achieved, respectively. PD was due to distant progression in 64/70 patients (91%) and to cutaneous progression with permeation nodes in 6/70 patients.

**Table 1:** Baseline patient and disease characteristics

Median age at inclusion (years)	66	[39–85]
Performance status		
0	39	37.9%
1	46	44.7%
2	11	10.7%
3	2	1.9%
NA	3	2.9%
BMI (kg/m^2^)	27	[18.3–56.5]
HER2 status		
Positive	9	8.7%
Negative	90	87.4%
NA	4	3.9%
Disease presentation at metastatic setting*		
De novo	53	51.4%
Relapsed	50	48.6%
Adjuvant treatment		
Chemotherapy	37	74.0%
Hormonotherapy	44	88.0%
Tamoxifen	34	77.3%
AI	25	56.8%
Median delay from end of adjuvant treatment to metastatic diagnostic (months)	57.5	[37–107]
Metastatic treatment before AI introduction		
Chemotherapy		
Yes	26	25.2%
No	77	74.8%
Endocrine therapy except AI		
Yes	13	12.6%
No	90	87.4%
AI status at inclusion		
Initiation at inclusion	75	72.8%
Already started without progression	28	27.2%
Median delay since AI introduction (months)	9.9	[6.2–63.8]
Median follow-up (months)	25.3	[3–92]

Data are presented as no. (%) unless indicated otherwise

*Presentation of advanced disease is defined as de novo (advanced at first presentation) or relapsed (relapsed after previous presentation with early-stage cancer)

### Biomarker analyses

A total of 596 blood samples were analyzed during the study period corresponding to a median of 6 consecutive samples (range 2–9) per patient. Circulating *ESR1* mutations were detectable at baseline in 4 patients: 1 already under AI treatment and 3 who initiated AI treatment at inclusion.

### Biomarker variations and correlation with progression

#### Circulating *ESR1* mutations

**Fig. 1:**
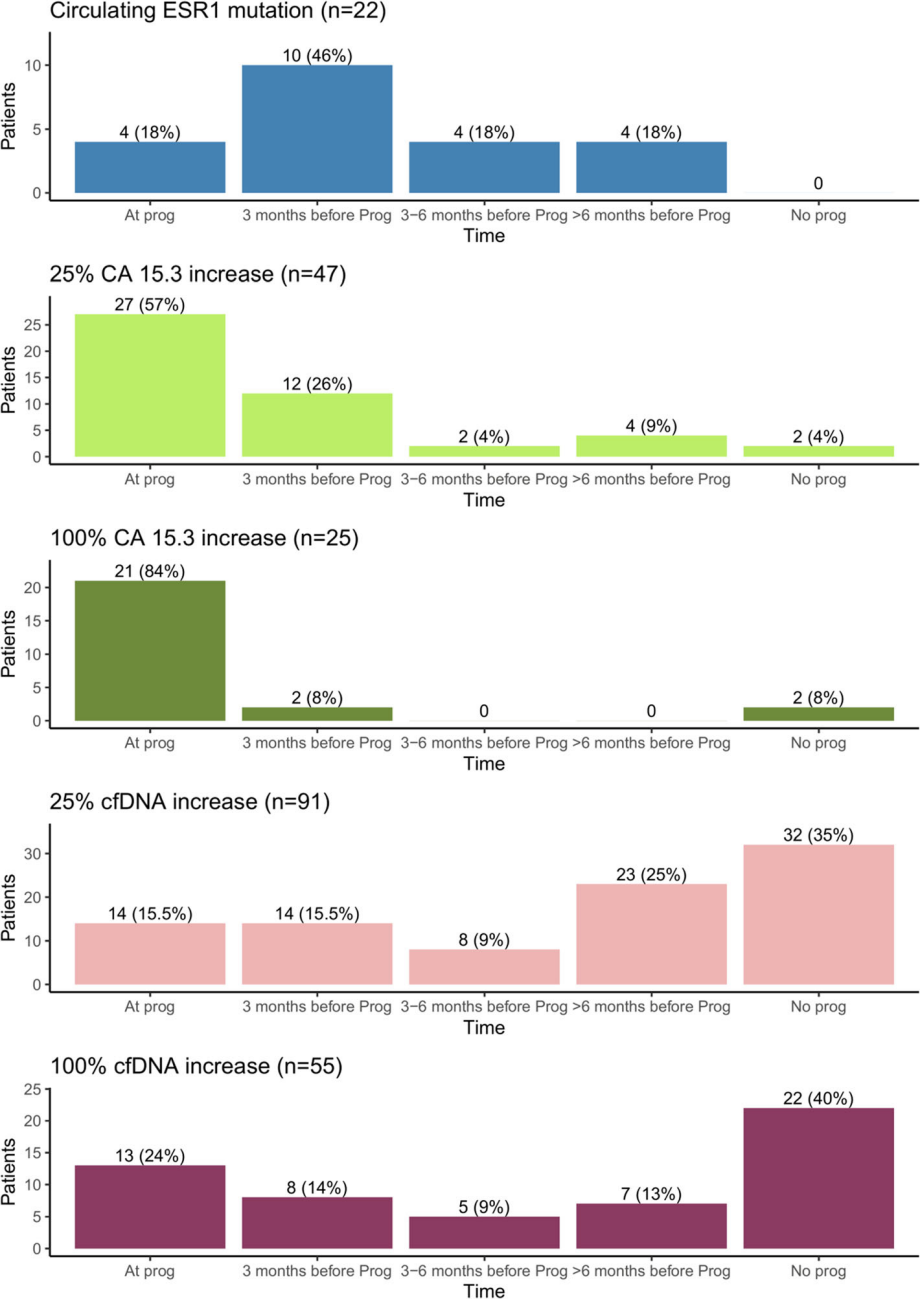
Correlation between biomarker variations and time to progression. For each biomarker, the number of patients with a biomarker event (biomarker increase or mutation emergence) according to the time of progression (PD) is reported

Among the 70 patients who experienced PD during follow-up, circulating *ESR1* mutations were detectable in 22 patients (31.4%), including 18/22 (82%) before PD and 4/22 (18%) at the time of progression (Fig. 1). *ESR1* mutation detection rate was not different between patients with de novo metastatic disease (11/53) and patients who relapsed after adjuvant treatment (11/50). Among the 22 patients with *ESR1* mutations detected at or before PD, 15 patients (68%) had a single mutation detected, while 7 patients had a polyclonal mutation. Among the 7 mutations tested, the D538G was the most frequent mutation, detected in 12/22 patients (55%). The detection rates for the other mutations were Y537S (10/22, 45%), Y537N (7/22, 32%), E380Q and Y537C (3/22, 14%), S463P (1/22, 5%), and L536R (0/22). *ESR1* mutation detection was significantly associated with PD (*p* < 0.001, chi-square test), without detectable mutations at any time in patients without PD. Overall, the median time from *ESR1* mutation detection to progression was 91 days [0–282] (Table 2).

**Table 2:** Incidence of biomarker variations and correlations with progression

Marker	All population		Progression	No progression		
	*n* = 103	%	*n* = 70	*n* = 33	*p*	Median delay (days)
*ESR1*						
*Mutated*	22	21%	22 (31%)	0 (0%)	< 0.001	91 [0–282]
*Non mutated*	81	79%	48 (69%)	33 (100%)		
> 25% CA-15.3 increase						
*Yes*	47	46%	45 (64%)	2 (6%)	< 0.001	0 [0–543]
*No*	56	54%	25 (36%)	31 (94%)		
> 100% CA-15.3 increase						
*Yes*	25	24%	23 (33%)	2 (6%)	0.003	0 [0–91]
*No*	78	76%	47 (67%)	31 (94%)		
> 25% DNA increase						
*Yes*	91	88%	59 (84%)	32 (97%)	0.1	182 [0–635]
*No*	12	12%	11 (16%)	1 (3%)		
> 100% DNA increase						
*Yes*	55	53%	33 (42%)	22 (67%)	0.1	92 [0–474]
*No*	48	47%	37 (58%)	11 (33%)		

*p* values were determined using a chi-square test

#### *ESR1* mutation detection before progression

Among patients with the emergence of circulating *ESR1* mutations before PD (*n* = 18), the median delay was 110 days (range 50–282) from circulating *ESR1* mutation detection to PD. The mutation was detected in every interval sample until progression in 15/18 of the patients (83%). Among the 3 patients with *ESR1* mutation at the initiation of AI treatment (baseline), one had a clearance of the mutation from month 3 to PD observed at month 9. Another patient had a continuous increase of the *ESR1* mutation value every 3 months until PD at month 6. Finally, the third patient had a decrease of the mutation level detected between baseline and month 3, while PD occurred at month 3. The presence of a circulating *ESR1* mutation was significantly associated with the risk of PD at the next follow-up with a RR of 4.9 [3.0–8.0] at 3 months and 3.3 [2.4–4.5] at 6 months and an overall RR of 1.9 [1.7–2.0] compared to patients without *ESR1* mutation detection (*p* < 0.001 in each case, Table 3).

**Table 3:** Risk ratio (RR) of progression according to each biomarker

Marker	Progression			No progression	Total
	≤ 3 months	≤ 6 months	Anytime after marker appearance		
ESR1 mutation					
*n* (%)	10 (56%)	14 (78%)	18 (100%)	0 (0%)	18 (100%)
RR (ref = non mut)	4.9 [3.0–8.0]	3.3 [2.4–4.5]	1.9 [1.7–2.0]	–	
> 25% CA-15.3 increase					
*n* (%)	12 (60%)	14 (70%)	18 (90%)	2 (10%)	20 (100%)
RR (ref = no increase)	5.9 [3.8–9.2]	3.4 [2.5–4.8]	2.0 [1.7–2.4]	–	
> 100% CA-15.3 increase					
*n* (%)	2 (50%)	2 (50%)	2 (50%)	2 (50%)	4 (100%)
RR (ref = no increase)	4.0 [1.5–11.0]	2.2 [0.8–5.8]	1.1 [0.4–2.8]	–	
> 25% DNA increase					
*n* (%)	14 (18%)	22 (29%)	45 (58%)	32 (42%)	77 (100%)
RR (ref = no increase)	1.6 [0.8–3.3]	1.1 [0.7–1.7]	0.9 [0.7–1.2]	–	
> 100% DNA increase					
*n* (%)	8 (19%)	13 (31%)	20 (48%)	22 (52%)	42 (100%)
RR (ref = no increase)	1.4 [0.7–2.7]	1.1 [0.6–1.7]	0.8 [0.6–1.2]	–	

#### CA-15.3 increase during follow-up

At baseline, median CA-15.3 value was 41 kUI/L [6–2454], and 61 patients (59%) had a supranormal CA-15.3 value (> 30 kUI/L). During follow-up, a CA-15.3 increase at a threshold of 25% was observed in 47/70 (67%) patients with PD, including 27 (57%) at the time of PD and 20 (43%) before (Fig. 1). Among patients without PD, a 25% CA-15.3 increase was observed in 2 patients (6%) (Table 2). A CA-15.3 increase at a threshold of 100% was observed in 23 (33%) patients with PD, including 21 (84%) at the time of PD and 2 (16%) before (Fig. 1). Among the patients without PD, a 100% CA-15.3 increase was observed in 2 patients (6%) (Table 2). Both CA-15.3 increases of 25% and 100% were correlated with PD (*p* < 0.001, chi-square test) (Table 2), with a median delay of 0 days from CA-15.3 increase to PD for both thresholds.

When a CA-15.3 increase of 25% was detected before progression, a median delay of 91 days [14–543] was observed from CA-15.3 increase to PD. The presence of a CA-15.3 increase of 25% was significantly associated with the risk of PD at the next follow-up with a RR of progression of 5.9 [3.8–9.2] at 3 months and 3.4 [2.5–4.8] at 6 months and an overall RR of 2.0 [1.7–2.4] compared to patients without a CA-15.3 increase (*p* < 0.001 in each case, Table 3). Of note, a CA-15.3 increase of 100% was not associated with a significant RR of progression (chi-square test), probably because of the low number of patients with this threshold (*n* = 4) (Table 3).

#### cfDNA increase during follow-up

A cfDNA increase of 25% was observed in 59 patients (84%) with PD, including 45 (76%) before and 14 (34%) at the time of progression (Fig. 1). Among patients without PD, a 25% cfDNA increase was observed in 32 patients (96%). A threshold of cfDNA increase at 100% was identified in 33 (47%) patients with PD, including 20 (61%) before and 13 (39%) at progression (Fig. 1). Among patients without PD, a 100% cfDNA increase was observed in 22 patients (67%). cfDNA increases of 25 or 100% were not significantly correlated with progression (chi-square test, Table 2). Overall, the presence of an increase in cfDNA using both thresholds was not associated with the risk of early PD (Table 3).

#### Prognostic value of biomarkers at progression on AI

**Fig. 2:**
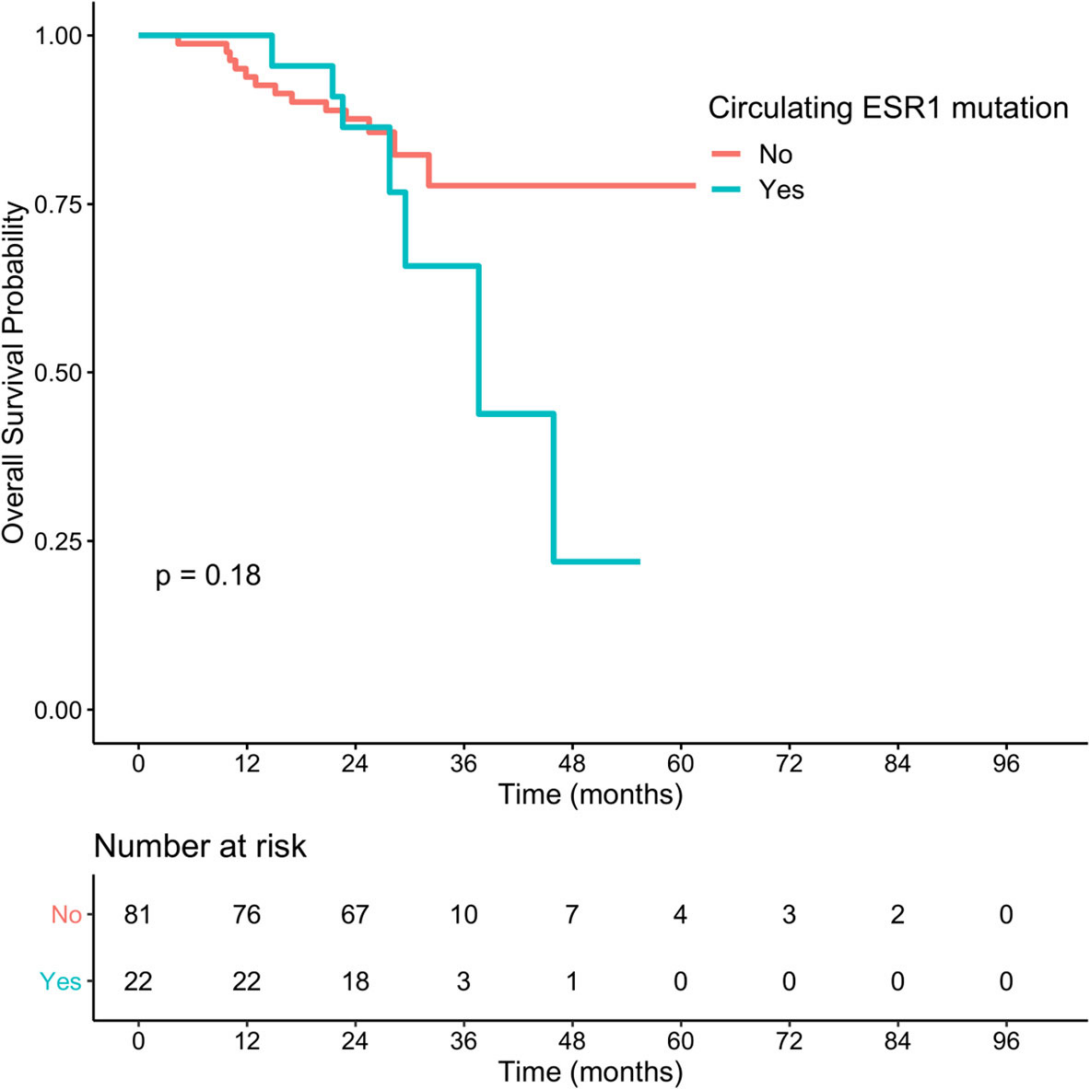
Overall survival according to ESR1 mutation status at progression disease. *p* value was determined using a log-rank test

**Fig. 3:**
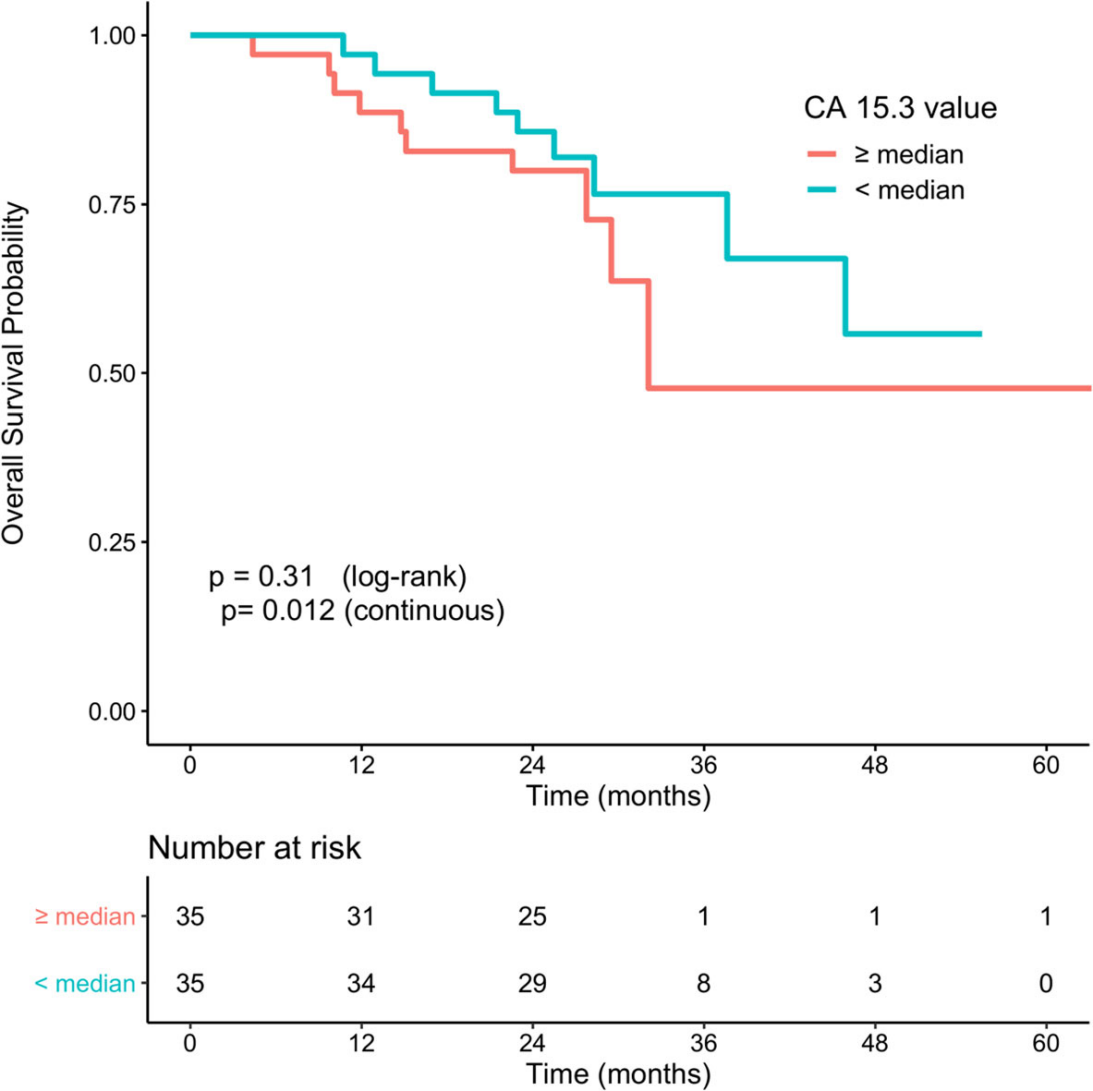
Overall survival according to CA-15.3 level at progression disease. *p* value was determined using a log-rank test or a Cox model

**Fig. 4:**
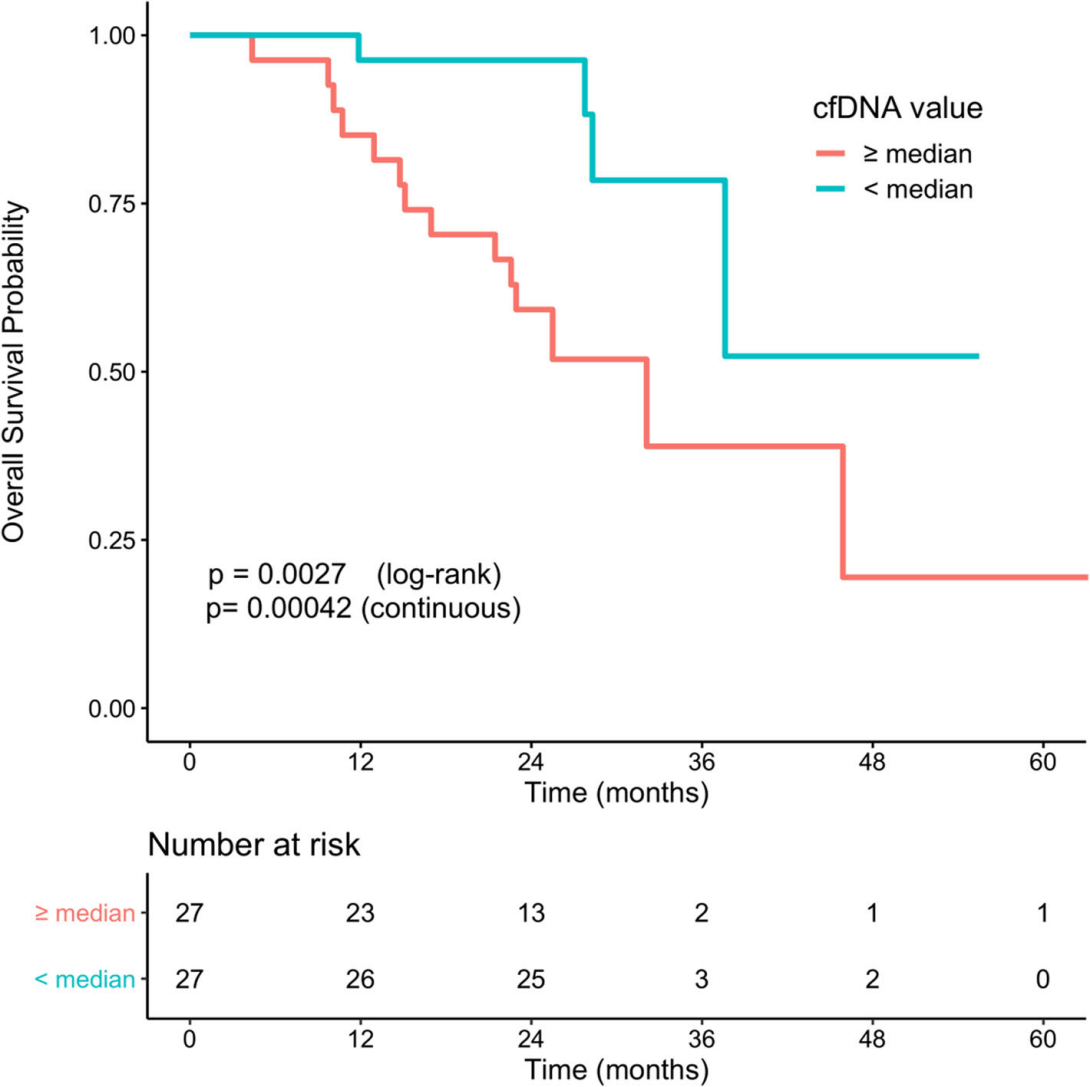
Overall survival according to cfDNA level at progression disease. *p* value was determined using a log-rank test or a Cox model

Among the 75 patients with the initiation of AI treatment at inclusion, the median CA-15.3 and median cfDNA were not correlated with PFS or OS (see supplementary). At progression on AI, and considering the low number of patients concerned, having a detectable circulating *ESR1* mutation was not associated with a worse outcome (Fig. 2). Elevated CA-15.3 was related to a worse OS only when regarded as a continuous variable (Fig. 3), while cfDNA values at PD were related to a significantly worse outcome when used as a median value and continuous variable (Fig. 4).

## Discussion

Our prospective study showed that the emergence of circulating *ESR1* mutations is associated with a 4.9- and 3.3-fold increase in the risk of PD at 3 and 6 months, respectively, in comparison to patients without *ESR1* mutations during AI treatment in HR+MBC. While CA-15.3 was also significantly associated with an increased risk of PD, with a 5.9- and 3.4-fold increase at 3 and 6 months, respectively, our findings support that *ESR1* monitoring is a better predictor than CA-15.3 in that setting. Indeed, we observed that circulating *ESR1* mutations occurred in almost 75% of patients before clinical progression, in contrast to the CA-15.3 increase, which occurred in 57% of patients concomitantly with PD. To our knowledge, these results have never been reported so far and *ESR1* tracking appears to date as the most clinically relevant marker for AI monitoring in HR+MBC.

As previously reported, circulating *ESR1* mutations were found in 31.4% of patients at progression, with a 82% detection rate before PD [8, 14]. The most frequent mutation identified was D538G, and polyclonal mutations were frequently observed (32%), as previously reported [8, 17]. To our knowledge, only one prospective study has evaluated the potential interest of *ESR1* mutations in predicting clinical progression under AI treatment. In this study, Fribbens et al. followed 72 patients under first-line AI treatment and found an emergence of circulating *ESR1* mutations in 22 of the 39 (56%) patients with PD. As in our work, these circulating mutations were present before progression in 19/22 patients (86%), with a median delay between the first circulating identification and PD of 6.7 months (range 3.7-NA). Our results are similar with 82% of circulating *ESR1* mutations occurring before progression, with a slightly shorter median delay of 3.7 months [1.7–9.4]. Moreover, we have shown that all patients with detectable circulating *ESR1* mutations progressed. In contrast, Fribbens et al. reported that 5/33 (15%) of the patients who did not progress had a detectable *ESR1* mutation without details regarding their clinical follow-up. To our knowledge, our study was the first to specifically quantify the risk of PD when *ESR1* mutations occurred, with 4.9-fold and 3.3-fold increases at 3 and 6 months of follow-up, respectively. Interestingly, the ongoing phase III randomized trial PADA-1 (NCT03079011) is evaluating the potential value of early treatment modification in the case of emergent *ESR1* mutation determined by ddPCR. Of note, *ESR1* detection by ddPCR in daily practice could be easily implemented in a molecular laboratory since detection kits are commercialized. The main issue is the delay limited to few hours between sample collection and process when using EDTA tubes. But the use of PAXgene or Streck tubes allows cfDNA preservation at room temperature up to 7 days between sample collection and first centrifugation [18].

When considering CA-15.3, the results were not different regarding the 25% or 100% increase thresholds. We also found that there was a significant association between CA-15.3 increase and PD, with a majority of cases (57%) occurring concomitantly with progression. Until now, CA15.3 remains the most frequent marker used in HR+MBC. Indeed, in previous studies focusing on the usefulness of CA-15.3 or carcinoembryonic antigen (CEA) to predict outcome, CA-15.3 is considered the best single biomarker in that setting [19–22]. The interest of biomarker combination including namely CA15.3 and CEA has been reported [23], even if the data remains conflicting with other work suggesting no increase of sensitivity and a decrease of the positive predictive value when considering a combination instead of CA-15.3 alone [19]. In our study, and in contrast to cfDNA assessment, CA 15.3 elevation was highly correlated to clinical evolution, and only 2 patients had a CA 15.3 increase > 25% without PD in the next months. However, it is noteworthy that since our design was based on a current follow-up of patients every 12 weeks, we have specifically planned the serum CA15.3 marker collection on the same schedule. Considering that 1-month sampling interval of CA15.3 is commonly used in the scientific literature pursuing the same aim in the same setting, the sampling interval of 12 weeks for serum CA15.3 maybe represents a limitation of this work [23–25].

Regarding cfDNA, an increase (either using 25% or 100% threshold) was not correlated with progression. Moreover, most of the patients without progression had previously experienced a cfDNA increase. Even if we found that elevated cfDNA at progression was associated with poor prognosis, the lack of a correlation between cfDNA variation and clinical progression makes this biomarker unsuitable for daily practice. A high level of cfDNA has been previously related to OS at progression in MBC patients [4, 8, 26, 27], but to our knowledge, this is the first study that investigated the potential use of cfDNA to predict progression. Even if the total amount of cfDNA is correlated with tumor stage, many mechanisms other than tumor progression may lead to an increase in cfDNA, such as necrosis, autophagy, and hypoxia [28], which may explain the lack of correlation between cfDNA variations and PD. Recently, the results of a study comparing the correlation between CA-15.3, cfDNA, CTCs, and alkaline phosphatase values in 194 MBC patients receiving various treatments, or not, were reported . The authors observed that cfDNA and CTCs were correlated with overall survival (HR 1.2 for both biomarkers), while cfDNA was the only biomarker correlated with progression-free survival. They concluded that a single cfDNA analysis could be an interesting biomarker for treatment evaluation in MBC patients. Their results are not comparable to ours since the population included was different, and since they considered an absolute value for each biomarker rather than a variation. Nevertheless, the poor AUC (0.593) that they observed when using cfDNA to discriminate patients who have responded or not makes this biomarker hard to use in daily practice for treatment adaptation [29].

This study has several limitations. First, 28/103 patients (27%) were already under AI treatment without progression at inclusion in this study, and we cannot exclude that a biomarker variation occurred in the first months of AI exposure. Of note, only one patient out of these 28 had a circulating *ESR1* mutation detected at inclusion. This mutation remained detectable in every 3-month sample until PD 9 months after inclusion. Second, this study was conducted before the combination of cdk4/6 inhibitors and AI was established as a first-line therapy for HR+MBC. Thus, our results may not be applicable to patients with AI+cdk4/6 inhibitors and dedicated studies are warranted in this population. On the other hand, recent results from the PALOMA-3 study revealed that *ESR1* mutation was still an important mechanism of endocrine therapy resistance under treatment with cdk4/6 inhibitors, with a peculiar selection of the *ESR1* Y537S mutation enrichment when patients are exposed to fulvestrant + palbociclib [30]. Third, in addition to circulating *ESR1* mutations that are associated with resistance to AI, other genomic alterations can be used to determine the amount of circulating ctDNA, such as *PIK3CA* mutations [17]. Due to the limited availability of plasma samples, we were unable to explore circulating molecular alterations other than *ESR1* mutations. Besides the use of ctDNA in the detection of PD in advanced breast cancer, ctDNA has also been investigated in early breast cancer to predict relapse after the end of adjuvant treatment. Garcia-Murillas et al. recently reported a lead time between ctDNA detection and relapse of 10.7 months (95% CI [8.1–19.1]) using a personalized ddPCR assay, and Coombes et al. reported a comparable lead time of 8.9 months (range 0.5–24) using NGS [31, 32]. Thus, it seems that ctDNA detection in the early setting may provide a longer lead time before relapse than for prediction of PD in the metastatic setting. Due to the limited data, no definitive conclusions can be drawn, but we may hypothesize that the higher tumor burden in the metastatic setting may lead to a shorter lead time between ctDNA detection and clinical progression.

## Conclusions

The present prospective study led to the quantification of the risk of early PD when circulating *ESR1* mutations emerge under AI treatment in HR+MBC, with an increase in the risk of progression of 4.9-folds at 3 months and 3.3-folds at 6 months compared to patients without *ESR1* mutations. We also highlighted that *ESR1* tracking was more relevant than the CA-15.3 increase that occurred in the majority of cases concomitantly with PD. Taken together, these results prompt the evaluation of a novel strategy of treatment based on circulating *ESR1* detection.

## Supplementary information

**Additional file 1.** Provides the following survival curves: PFS according to cfDNA level at baseline. OS according to cfDNA level at baseline. PFS according to CA-15.3 level at baseline. OS according to CA-15.3 level at baseline.

## Data Availability

The datasets used and/or analyzed during the current study are available from the corresponding author on reasonable request.

## Abbreviations

AI: Aromatase inhibitor
CEA: Carcinoembryonic antigen
cfDNA: Cell-free DNA
CTC: Circulating tumor cell
ddPCR: Digital droplet polymerase chain reaction
*ESR1*: Estrogen receptor gene
HR+: Hormone receptor positive
MBC: Metastatic breast cancer
NGS: Next-generation sequencing
OS: Overall survival
PD: Progressive disease
PFS: Progression-free survival
TPA: Tissue polypeptide antigen
VAF: Variant allele fraction
