# Supplementary material for: Risk of early progression according to circulating ESR1 mutation, CA-15.3 and cfDNA increases under first-line anti-aromatase treatment in metastatic breast cancer
Source: Breast Cancer Res. 2020 May 28;22:56. doi: 10.1186/s13058-020-01290-x (PMC7254698; doi:10.1186/s13058-020-01290-x)
Supplement: Supplementary file 1 — Additional file 1. Provides the following survival curves: PFS according to cfDNA level at baseline. OS according to cfDNA level at baseline. PFS according to CA-15.3 level at baseline. OS according to CA-15.3 level at baseline. [file 13058_2020_1290_MOESM1_ESM.docx]

Supplementary


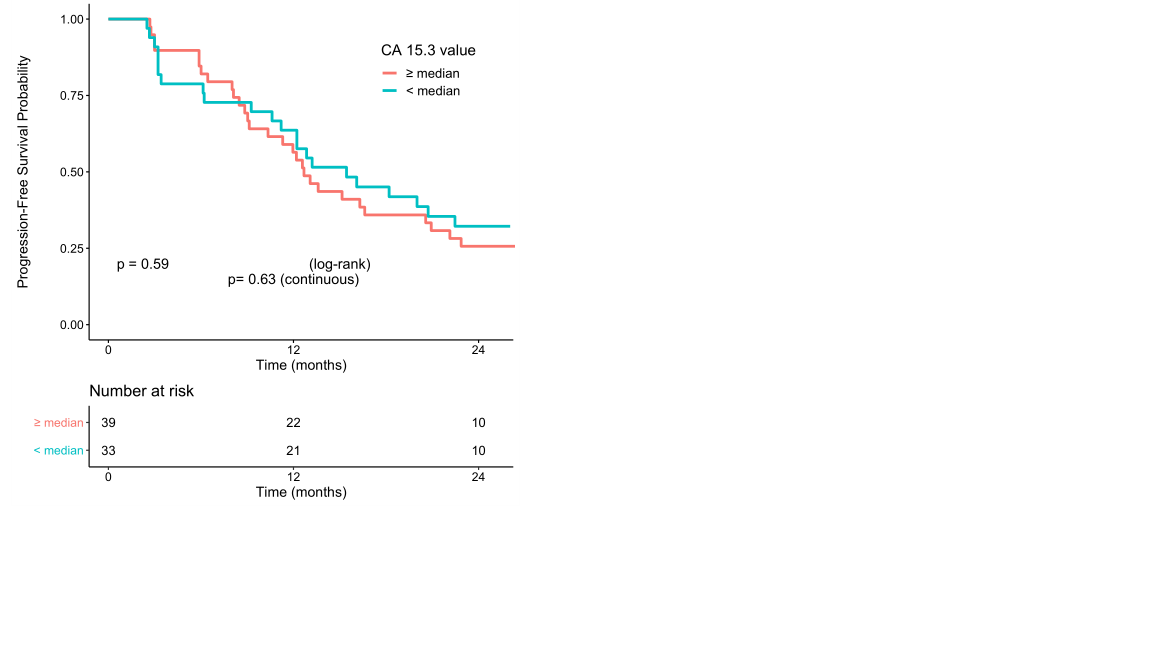


**Progression free survival according to CA-15.3 level at baseline**

p values were determined using a log-rank test or a Cox model


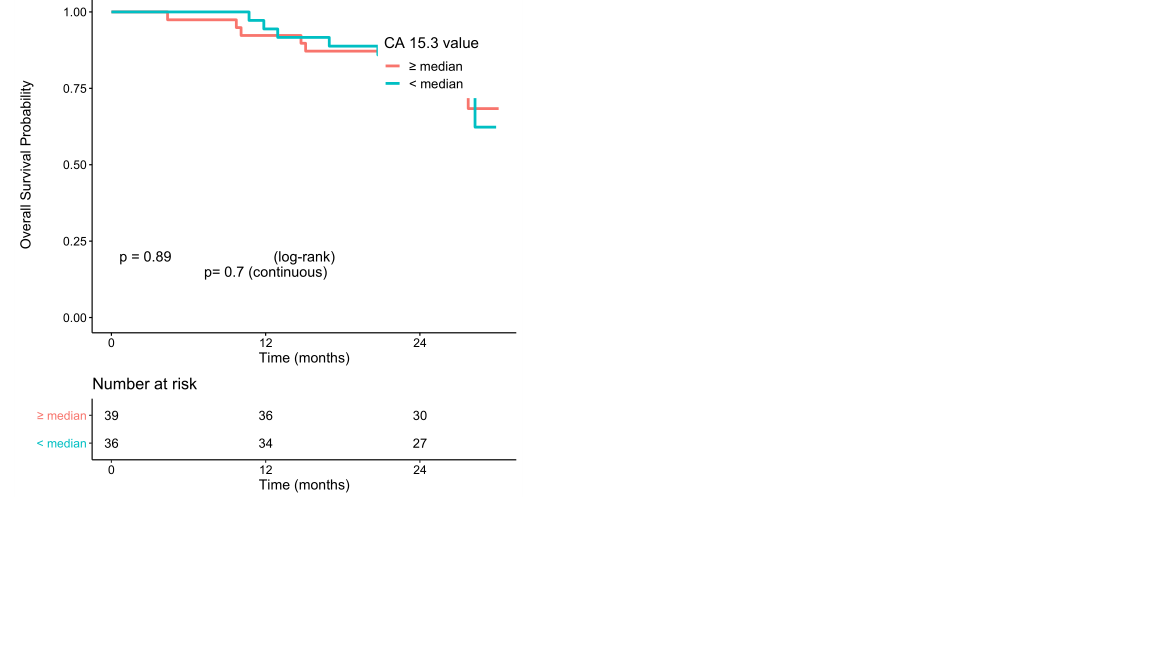


**Overall survival according to CA-15.3 level at baseline**

p values were determined using a log-rank test or a Cox model


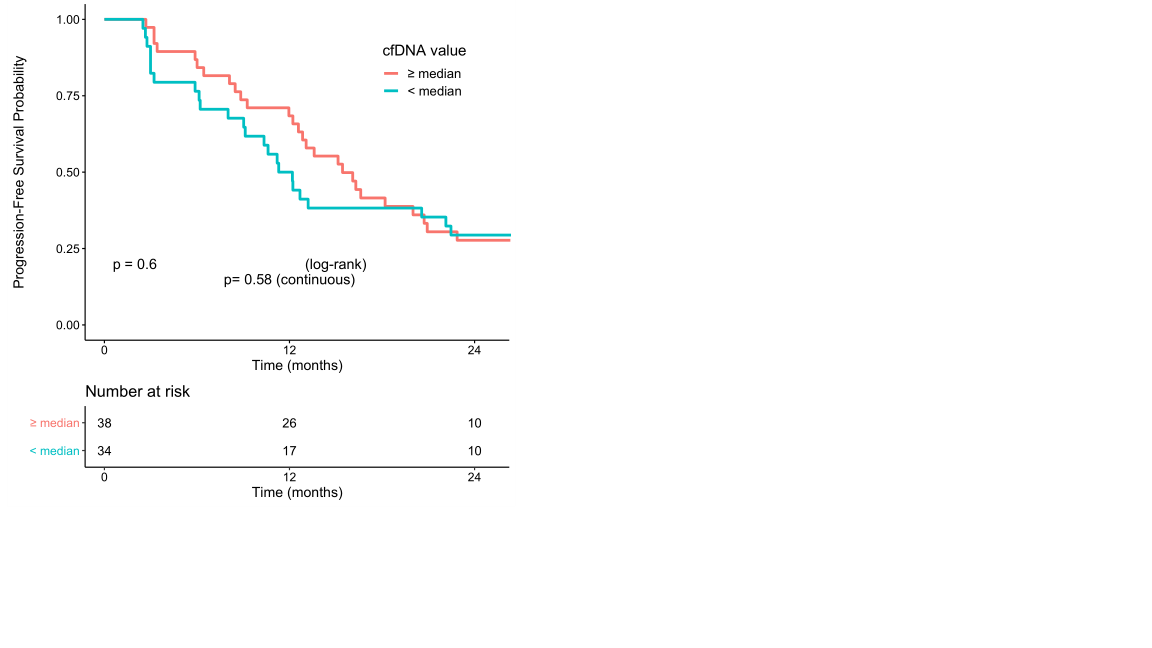


**Progression free survival according to cfDNA level at baseline**

p values were determined using a log-rank test or a Cox model


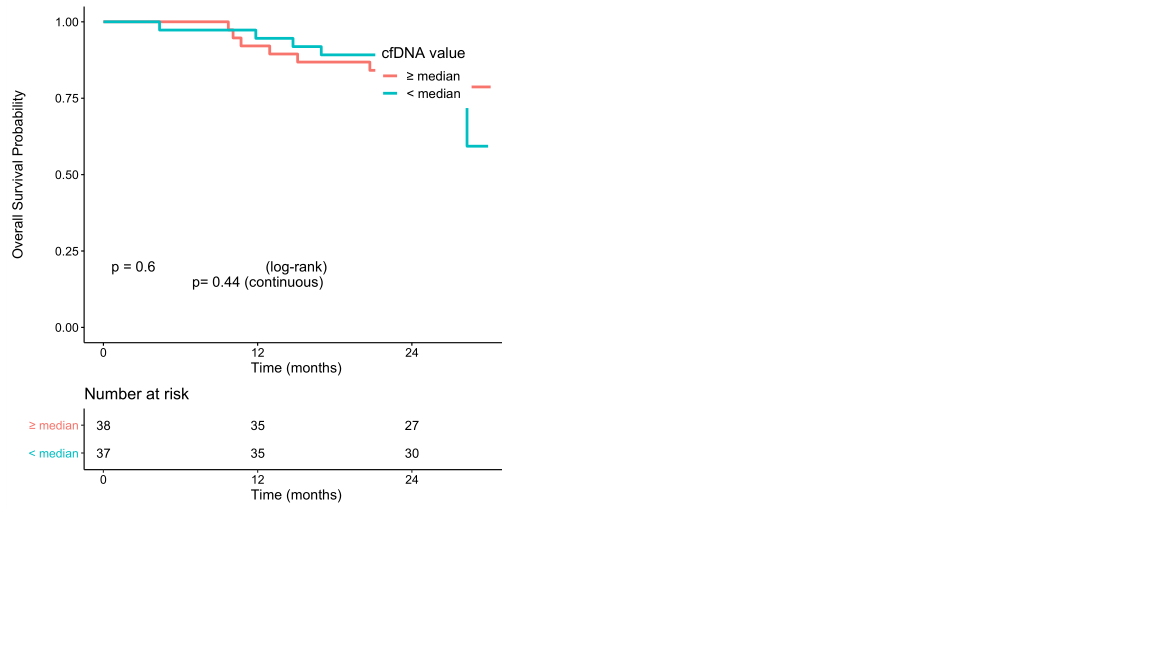


**Overall survival according to cfDNA level at baseline**

p values were determined using a log-rank test or a Cox model
